# Successful Treatment of TAFRO (Thrombocytopenia, Anasarca, Fever, Renal Insufficiency, and Organomegaly) Syndrome With Triple Combination Therapy of Corticosteroid, Tocilizumab, and Cyclosporine: A Case Report

**DOI:** 10.7759/cureus.80274

**Published:** 2025-03-08

**Authors:** Junki Morino, Keiji Hirai, Katsuyuki Yoshida, Shinichi Kako, Susumu Ookawara, Hisashi Oshiro, Hitoshi Sugawara, Yoshiyuki Morishita

**Affiliations:** 1 Division of Nephrology, First Department of Integrated Medicine, Jichi Medical University Saitama Medical Center, Saitama, JPN; 2 Division of General Medicine, First Department of Integrated Medicine, Jichi Medical University Saitama Medical Center, Saitama, JPN; 3 Division of Hematology, First Department of Integrated Medicine, Jichi Medical University Saitama Medical Center, Saitama, JPN; 4 Department of Diagnostic Pathology, Jichi Medical University Saitama Medical Center, Saitama, JPN

**Keywords:** acute renal failure, corticosteroid, cyclosporine, tafro syndrome, tocilizumab

## Abstract

We report a case of the most severe grade TAFRO (thrombocytopenia, anasarca, fever, renal insufficiency, and organomegaly) syndrome that was successfully treated with triple combination therapy of corticosteroid, tocilizumab and cyclosporine. A 48-year-old man with a history of thrombotic thrombocytopenic purpura was referred to our department for dyspnea, pleural effusion, and ascites. Further examination revealed thrombocytopenia (platelet count 4.7×10^4^/μL), severe renal dysfunction (creatinine 2.56 mg/dL), and systemic inflammation (C-reactive protein 18.52 mg/dL). Computed tomography revealed mild lymphadenopathy and mild hepatomegaly. Bone marrow examination showed hypocellularity and increased reticulin fibrosis, and lymph node biopsy showed proliferation of endothelial venules. Thus, the patient was diagnosed with TAFRO syndrome. After the combination of steroid pulse therapy (intravenous methylprednisolone 1 g/day for three days and subsequent oral prednisolone 60 mg/day), tocilizumab at 480 mg once weekly, and cyclosporine at 225 mg once daily, systemic inflammation and kidney function gradually improved. This case suggests that the triple combination therapy can lead to remission of the most severe grade TAFRO syndrome.

## Introduction

TAFRO (thrombocytopenia, anasarca, fever, renal insufficiency, and organomegaly) syndrome is a systemic disease characterized by thrombocytopenia, fluid retention including pleural effusion and ascites, fever, systemic inflammation, kidney dysfunction, and organomegaly such as hepatomegaly and splenomegaly [1]. The disease can lead to irreversible organ damage and death. Although TAFRO syndrome is a new disease definition, several immunosuppressive drugs have been reported to improve organ dysfunction and prognosis [2-4]. It is suggested that the therapeutic effect is mediated by suppressing inflammatory cytokines with corticosteroids, suppressing interleukin (IL)-6-mediated cascades with tocilizumab, and suppressing T cells and steroid resistance with cyclosporine. The efficacy of corticosteroids, tocilizumab, and cyclosporine as monotherapy or dual combination therapy for TAFRO syndrome has been demonstrated [4]. However, the efficacy of their triple combination therapy in severe grade TAFRO syndrome has not yet been reported. We report a case of the most severe grade TAFRO syndrome that was successfully treated with a triple combination therapy of corticosteroid, tocilizumab, and cyclosporine.

## Case presentation

A 48-year-old man was referred to our department with a two-week history of dyspnea, pleural effusion and ascites, worsening renal function, and C-reactive protein (CRP) elevation. He had a past history of thrombotic thrombocytopenic purpura (TTP) treated with plasma exchange and corticosteroid six years previously. Drug therapy had been terminated due to TTP remission four years ago. His renal function was managed within the normal range with creatinine 0.85 mg/dL two months ago. 

**Figure 1:**
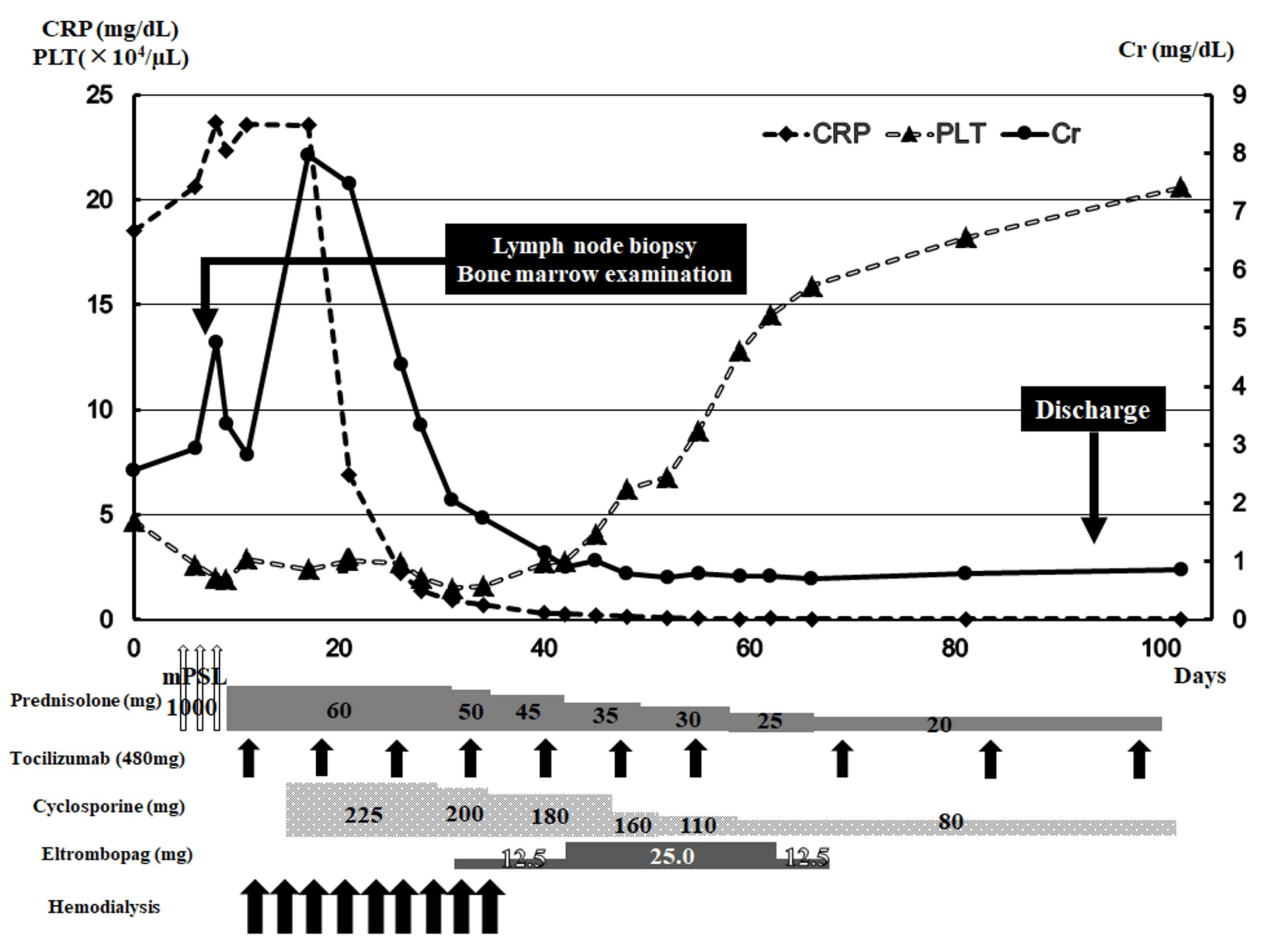
Clinical course of the patient. The horizontal axis shows the number of days from admission. The vertical axes show the CRP, PLT, and Cr levels. Cr, creatinine; CRP, C-reactive protein; mPSL, methylprednisolone; PLT, platelets.

On admission, physical examination revealed trunk and lower limb edema, and there was no erythema, urticaria, pruritic edema, or tenderness suggestive of insect bites. His blood pressure was 103/67 mmHg, pulse rate 89 beats/minute, body temperature of 37.7℃, respiratory rate of 14 beats/minute, blood oxygen saturation level of 93% with room air, and Glasgow Coma Scale E4V5M6. Blood analysis showed thrombocytopenia (platelet count 4.7×10^4^/μL), severe renal dysfunction (creatinine 2.56 mg/dL, blood urea nitrogen (BUN) 48 mg/dL), and systemic inflammation (CRP 18.52 mg/dL) (Figure 1). 

Serological tests for autoantibodies, including antinuclear antibodies, anti-neutrophil cytoplasmic antibodies, and anti-ADAMTS13 antibodies, blood culture tests, tuberculosis-specific interferon-γ tests, and anti-rickettsial antibody tests were all negative. There was no reduction in ADAMTS13 activity. Serum immunoglobulins, including IgG4, were within the normal range and no monoclonal protein was detected. A detailed description of the laboratory data at that time is shown in Table 1. 

**Table 1:** Laboratory results at the time of referral to our department ANCA, anti-neutrophil cytoplasmic antibody; BJP, Bence–Jones protein; C3, complement component 3; C4, complement component 4; CH50, 50% hemolytic unit of complement; DNA, deoxyribonucleic acid; eGFR, estimated glomerular filtration rate; GBM, glomerular basement membrane antibody; HbA1c, hemoglobin A1c; HDL, high-density lipoprotein; HPF, high-power field; IFN, Interferon; IgA, immunoglobulin A; IgG, immunoglobulin G; IgM, immunoglobulin M; IL-6, Interleukin-6; LDL, low-density lipoprotein; MPO, myeloperoxidase; PR-3, proteinase-3; sIL-2R, soluble interleukin-2 receptor; TSAT, transferrin saturation.

Examination	Patient values	Reference range
Blood tests		
White blood cells (/μL)	7400	3900-9800
Neutrophils (%)	87.0	40-74
Lymphocytes (%)	7.0	19-48
Monocytes (%)	6.0	3.4-9.0
Eosinophils (%)	0.0	0-7
Basophils (%)	0.0	0-2
Red blood cells (/μL)	502×10^4^	427-570×10^4^
Hemoglobin (g/dL)	14.9	12.0-17.6
Hematocrit (%)	46.5	39.8-51.8
Mean corpuscular volume	92.6	83-101
Platelets (×10^4^/μL)	4.7	13.0-36.9
Total protein (g/dL)	5.9	6.4-8.2
Albumin (g/dL)	1.8	3.9-5.1
Total bilirubin (mg/dL)	0.83	0.2-1.0
Aspartate aminotransferase (mU/mL)	49	11-30
Alanine aminotransferase (mU/mL)	31	4-30
Lactate dehydrogenase (mU/mL)	231	110-220
Creatine phosphokinase (IU/L)	45	30-190
Total cholesterol (mg/dL)	98	142-248
LDL-cholesterol (mg/dL)	60	<140
HDL-cholesterol (mg/dL)	10	48-103
Triglycerides (mg/dL)	142	30-117
Sodium (mEq/L)	137	138-145
Potassium (mEq/L)	4.3	3.6-4.8
Chloride (mEq/L)	101	100-110
Calcium (mg/dL)	10.2	8.6-10.1
Phosphate (mg/dL)	4.7	2.7-4.6
Blood urea nitrogen (mg/dL)	48	8-20
Creatinine (mg/dL)	2.56	0.65-1.07
eGFR (mL/min/1.73 m^2^)	22.8	≥60.0
C-reactive protein (mg/dL)	18.52	<0.20
Blood glucose (mg/dL)	133	70-100
Ferritin (ng/mL)	738.9	6.2-282.6
TSAT (%)	8.7	25-45
HbA1c (%)	6.3	4.6-6.2
IgG (mg/dL)	1377	870-1700
IgG4 (mg/dL)	37.4	4.5-117
IgA (mg/dL)	233	110-410
IgM (mg/dL)	48	33-190
C3 (mg/dL)	146	65-135
C4 (mg/dL)	30	13-35
CH50 (U/mL)	63.5	30.0-45.0
IL-6 (pg/mL)	36.0	≤7.0
sIL-2R (U/mL)	1198	122-496
Antinuclear antibody	40	≤40
PR3-ANCA (IU/mL)	<1.0	<1.0
MPO-ANCA (IU/mL)	<1.0	<1.0
Anti-GBM antibody (IU/mL)	<2.0	<3.5
Anti-double stranded DNA IgG (IU/mL)	<10	<10
Anti-platelet antibody	-	-
ADAMTS13 activity (IU/mL)	0.32	>0.10
ADAMTS13 inhibitor quantitative	<0.5	<0.5
Direct Coombs test	-	-
Indirect Coombs test	-	-
T-SPOT tuberculosis specific IFNγ	-	-
Urine tests		
pH	5.5	5.0-7.5
Specific gravity	1.020	1.005-1.025
Protein	1+	-
Glucose	-	-
Red blood cells (/HPF)	1-4	0-4
White blood cells (/HPF)	1-4	0-4
BJP	-	-
Monoclonal protein	-	-
Urinary protein excretion (g/g Cr)	0.13	<0.15

**Figure 2:**
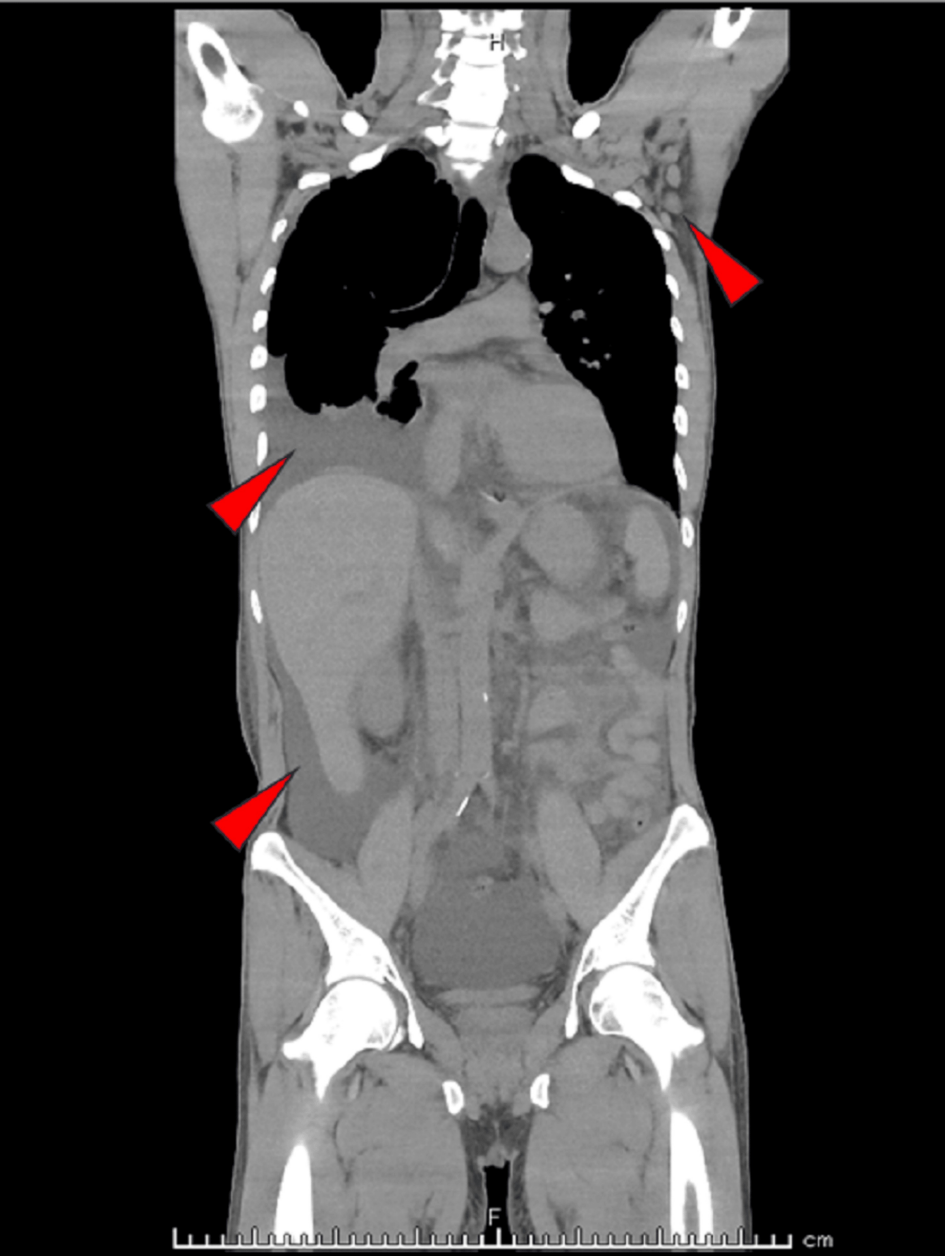
Coronal scan of computed tomography showing pleural effusion, ascites, and mild lymphadenopathy (red arrowheads).

**Figure 3:**
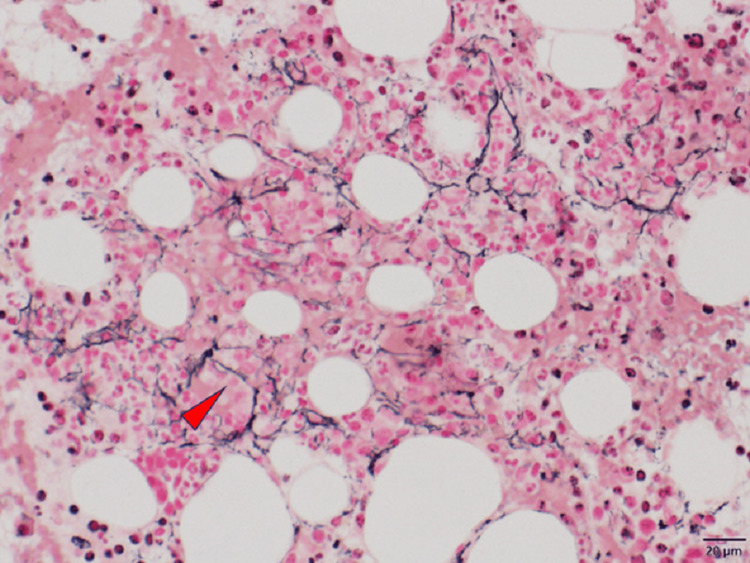
Bone marrow examination (sliver impregnation) showing hypocellularity and increased reticulin fibrosis (red arrowhead).

**Figure 4:**
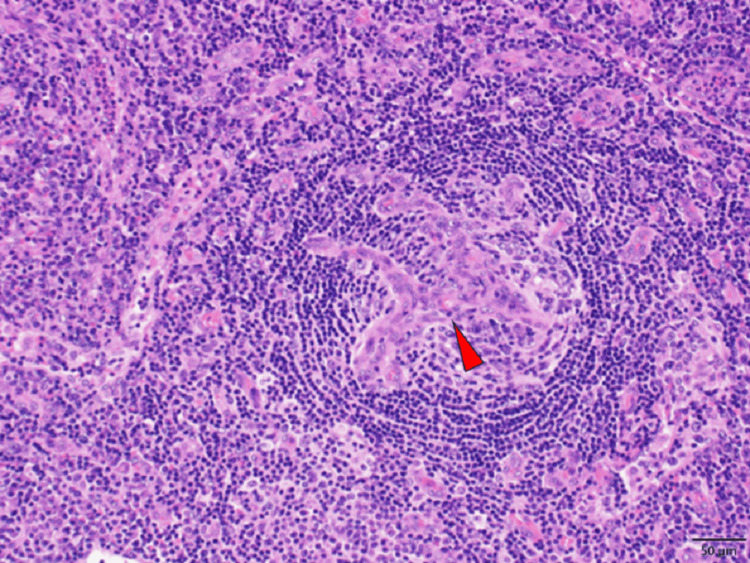
Lymph node biopsy showing proliferation of endothelial venules (red arrowhead).

Computed tomography revealed pleural effusion, ascites, mild lymphadenopathy, and mild hepatomegaly (Figure 2). On hospital day (HD) 7, he underwent a bone marrow examination which revealed increased reticulin fibrosis (Figure 3). Histological analysis revealed hypocellularity. Lymph node biopsy revealed proliferation of endothelial venules (Figure 4). These were compatible with the findings of Castleman disease [5,6]. There were no findings suggesting recurrence of TTP, malignancy, infectious disease, autoimmune disease, vasculitis, cirrhosis, POEMS (polyneuropathy, organomegaly, endocrinopathy, monoclonal plasma cell disorder, and skin changes) syndrome, or IgG4-related disease.

Based on his clinical course, he was diagnosed with TAFRO syndrome. Hence, steroid pulse therapy (intravenous methylprednisolone 1 g/day for three days) with subsequent oral steroid therapy (prednisolone 60 mg/day) was started. On HD 9, hemodialysis was initiated via a catheter because of worsening renal function (creatinine 4.76 mg/dL, BUN 145 mg/dL) (Figure 1), decreased urine volume (<100 mL/day), and volume overload. He was classified as the most severe disease by severity classification [1]. 

Tocilizumab (TCZ) 480 mg once weekly was started on HD 12 because of persistent systemic inflammation, residual pleural effusion and ascites, and poor response to steroid therapy (Figure 1). However, there was no improvement in thrombocytopenia and severe inflammation; therefore, cyclosporine 225 mg (5 mg/kg) once daily was started on HD 19 based on a previous report [7]​​​ (Figure 1). On HD 22, CRP level decreased from 23.55 to 6.88 mg/dL and urine output increased from anuria to 700-1000 mL /day (Figure 1). On HD 35, hemodialysis was discontinued because of improved renal function, confirmed by a 24-hour urine test (creatinine clearance 24.7 mL/min) (Figure 1). Because of the development of steroid psychosis and favorable response to the triple combination therapy, oral prednisolone was tapered by 5 mg per week. Subsequently, renal function and systemic inflammation were ameliorated. The dose frequency of TCZ was reduced to every two weeks after HD 54 (Figure 1). Eltrombopag olamine 12.5 mg once daily was started on HD 43, and ameliorated thrombocytopenia from 10,000-20,000 to 140,000-150,000/µL on HD 65 (Figure 1). 

The course of triple combination therapy was uneventful, and the patient was discharged on HD 70 (Figure 1). After outpatient care, white blood cell count and CRP level remained within normal ranges, and renal function was stable with no thrombocytopenia.

## Discussion

TAFRO syndrome was first reported in Japan in 2010 and is a systemic disease characterized by thrombocytopenia, anasarca, fever, kidney disfunction and organomegaly [1]. TAFRO syndrome is classified as a subtype of idiopathic multicentric Castleman disease [8]. We diagnosed this case of TAFRO syndrome based on pleural effusion and ascites, thrombocytopenia, and systemic inflammation as major categories, and lymphadenopathy, hepatomegaly, and progressive renal insufficiency as minor categories, and it was classified as grade 5 [1]. All markers suggestive of infectious disease, autoimmune disease, malignancies, hepatic cirrhosis, thrombocytopenic purpura, and hemolytic uremic syndrome were negative, which was compatible with the laboratory findings of TAFRO syndrome.

Because this case was classified as grade 5 and had a poor response to steroid therapy, steroids and TCZ were started. The lack of response to these treatments resulted in the initiation of cyclosporine in combination, which improved systemic inflammation and renal function. Steroid therapy has been shown to have beneficial effects on TAFRO syndrome; however, in some cases, steroid monotherapy may not be effective [9]. In previous studies, high hemoglobin and/or low CRP levels were predictors of response to steroid monotherapy [10]. In this case, the patient had a high level of systemic inflammation (CRP 23.55 mg/dL), and we initiated combination therapy with corticosteroid and TCZ. TCZ is a monoclonal antibody targeted against IL-6 receptor and is approved for the treatment of idiopathic multicentric Castleman disease in Japan, but not in Europe or the United States. In contrast, siltuximab, which is a monoclonal antibody targeted against IL-6, is approved in many countries but not in Japan. Thus, we started TCZ therapy. It is hypothesized that TCZ improves IL-6-induced autocrine lymph node hyperplasia by inhibiting IL-6, resulting in improvement of cachexia, systemic inflammation, and nutritional status [11]. 

The present case had the most severe grade of TAFRO syndrome; thus, we considered that corticosteroid and TCZ therapy were not sufficient to achieve rapid remission. Cyclosporine inhibits lymphocyte proliferation and differentiation by suppressing the production of cytokines, such as IL-2, IL-5, and interferon-γ through suppression of T cells [3]. Cyclosporine has also been reported to improve steroid resistance by antagonizing P-glycoprotein [12]. Although the detailed pharmacological mechanism of TAFRO syndrome is unclear, it has been suggested that remission can be achieved by improvement of lymph node hyperplasia and resolution of drug resistance. Further case accumulation is necessary to determine whether it is better to administer cyclosporine from the beginning for severe cases of TAFRO syndrome.

There are also reports that thrombocytopenia does not improve with immunosuppressive therapy alone but rather with the use of thrombopoietin receptor agonist [13]. After approximately one month of treatment with eltrombopag olamine, thrombocytopenia improved to within the normal range in the current case.

In this case, early use of triple drug combination therapy enabled induction of remission and withdrawal of dialysis, even in a grade 5 TAFRO syndrome. Thus, in the most severe cases, the importance of early initiation of combination therapy is suggested.

## Conclusions

We presented a case of the most severe grade TAFRO syndrome. The patient was successfully treated with triple combination therapy of corticosteroid, cyclosporin, and tocilizumab. Our case suggests that this triple combination therapy can lead to remission of the most severe grade TAFRO syndrome.
